# Botulinum Toxin Services for Neurorehabiliation: Recommendations for Challenges and Opportunities during the COVID-19 Pandemic

**DOI:** 10.3390/toxins13080584

**Published:** 2021-08-22

**Authors:** Ganesh Bavikatte, Jorge Jacinto, Thierry Deltombe, Joerg Wissel

**Affiliations:** 1 The Walton Centre NHS Foundation Trust, Liverpool L9 7LJ, UK; 2 Alcoitão Rehabilitation Medicine Centre, 2645-019 Alcabideche, Portugal; jor.jacinto@netcabo.pt; 3 Department of Physical Medicine & Rehabilitation, CHU UCL Namur Site Godinne, 5530 Yvoir, Belgium; thierry.deltombe@uclouvain.be; 4 Department of Neurorehabilitation and Physical Therapy, Vivantes Hospital Spandau, 13585 Berlin, Germany; joerg@schwarz-wissel.de

**Keywords:** botulinum toxin, muscle spasticity, dystonia, sialorrhea, COVID-19

## Abstract

The COVID-19 pandemic severely impacted the function of medical facilities and rehabilitation services worldwide, including toxin services delivering Botulinum toxin treatments for neuromuscular conditions such as spasticity, dystonia, and sialorrhea. The aim of this paper is to understand how toxin services have dealt with the situation and what strategies have been adopted to continue services. The recommendations are based on a virtual round table held with toxin services experts from different European countries who shared their experiences and discussed the best practices. The challenges for toxin services were reviewed based on the experts’ experiences and on relevant literature from 2020 and 2021. A set of recommendations and best practices were compiled, focusing firstly on guidance for clinical practice, including assessing patients’ health and risk status and the urgency of their treatment. Secondly, it was discussed how patients on botulinum toxin therapy can be cared for and supported during the pandemic, and how modern technology and tele-medicine platforms can be generally used to optimize effectiveness and safety of toxin treatments. The technological advances prompted by the COVID-19 crisis can result in better and more modern patient care in the future.

## 1. Introduction

The ongoing COVID-19 pandemic severely impacted the routine function of medical facilities and rehabilitation services worldwide [1,2,3], including toxin services delivering Botulinum toxin (BoNT) treatments for neuromuscular conditions. BoNT injections are the standard of care for focal spasticity [4,5] and dystonia [6,7]. They are also recommended for the treatment of sialorrhea (drooling), a common issue in various neurological conditions, including cerebral palsy, Parkinson’s disease, and stroke [8,9].

As a result of the pandemic, and the measures to combat it, toxin services around the world saw a significant reduction in patient visits and BoNT injections in 2020. While acute facilities, such as intensive care units (ICUs), were in the focus in the first months of the pandemic, out-patient facilities such as toxin clinics had to deal with closures and limitations to their services [10]. Now, in 2021, facilities have established a new way of working, and vaccination campaigns for staff and patients have been invaluable for resuming services. Telemedicine tools have seen an unprecedented boom [11], with new software becoming available and insurance reimbursement becoming more flexible, at least in some countries. Dealing with novel virus variants, further pandemic waves, and non-vaccinated staff and patients will now pose the next challenges, even in countries where the situation is deemed under control. Clinics will not be returning to pre-pandemic ways of working any time soon.

As BoNT therapy requires regular injections to maintain the clinical effects, a delay or prolonged interruption of therapy may have lasting negative impacts on patients’ health and quality of life. A survey from a German BoNT outpatient clinic during the first lockdown in spring 2020 showed delayed BoNT therapy by a mean of 6.6 weeks. As a result, >80% of their patients noticed an increase in muscle cramps and pain, quality of life was reduced by around 40%, and 98% of patients “perceived the shutdown as inadequate and felt their patient rights not respected” [12]. These data were later supported by a survey from Italy, using the same questionnaire. There, BoNT therapy was delayed notably more due to the lockdown, by a mean of 9 months. After that, 44% of patients had increased muscle cramps, quality of life was reduced by 68%, and 76% of patients “perceived the lockdown as inadequate and felt their patient rights not respected”. The survey additionally documented an increased burden on patients’ carers due to the lockdown [13]. Thus, toxin services urgently need to ensure appropriate and timely patient care. Another aspect that must be considered is the fear of a “wave” of out-patients with seriously deteriorated health due to suspended treatments, returning to rehabilitation services after the end of the pandemic, together with the emerging group of COVID-19 survivors who also often require rehabilitation [14,15].

The aim of this paper is to provide guidance and recommendations for toxin services to adjust to the “new normal”. We would like to share the clinical experience and perspective of leading experts from Europe in the area of neurorehabilitation. The authors are part of the ToxNet group (www.toxnet.net, accessed on 21 August 2021), a worldwide network of physicians with long-standing experience delivering toxin services to patients with spasticity, dystonia, or sialorrhea, aiming to establish solutions and best practices [5]. In the following, we detail our recommendations for continuous patient care and follow-up, and we give recommendations for optimizing the benefits of BoNT treatment. We would like to help turn the challenges into opportunities for modernization and for a fresh focus on patient needs.

## 2. Challenges and Recommendations

### 2.1. Challenges and Limitations for Toxin Services Due to the Pandemic

The COVID-19 pandemic has posed a number of challenges for patients and carers. In the acute first phase of the pandemic, the need to divert healthcare staff and resources to focus on the pandemic resulted in the reduction or complete suspension of toxin and therapy services in hospitals and out-patient facilities. Even where toxin services were still available or became available again, patients and carers may have experienced difficulties in access due to unavailable transport options. Moreover, the fear of infection and need for self-isolation may have discouraged patients from visiting their healthcare practitioner to obtain the necessary referrals for initiation of BoNT treatment or similarly discouraged them from continuing with BoNT treatment, when available.

Overall, large differences were observed between facilities regarding their ability to maintain treatment and follow-up, depending on whether a facility is a general hospital or a dedicated rehabilitation center or only an out-patient clinic without physiotherapy and occupational therapy services, and whether COVID-19 cases are present.

### 2.2. Recommendations for Clinical Practice

#### 2.2.1. Pre-Visit Patient Risk Assessment

Before a visit to a toxin services facility, a patient’s risk status should be assessed. Two separate aspects have to be considered: the patient’s risk of transmitting or contracting SARS-CoV-2, and the health risk if their neurological condition is not treated and their symptoms worsen (Table 1) [2,10]. The combination of these two assessments will inform the decision when to schedule a visit. Denying a patient an injection can be a very difficult decision, as patients are usually keen to be treated. Thus, the decision of postponing a visit should be explained to the patient in detail.

As patients may be anxious and worried about attending a facility for treatment, it can be helpful to provide them with a Q&A sheet answering the most important questions about how the visit and the procedures will take place, what precautions are in place, what the patient should expect to be different from before, and why [16].

Video communication can be highly useful to assess patients’ functional status, the impact of symptoms, and the urgency of treatment if face-to-face consultation is not possible [2,10,17,18,19]. If the toxin treatment has to be postponed, regular video calls are the best way of reviewing the patient’s coping strategies, i.e., to advise patients and carers regarding the management of symptoms and to assess the need for prescriptions of suitable medication (e.g., pain medication, muscle relaxants). Such tele-support can lead to more flexible treatment intervals in the long run. It is our opinion that it would be advantageous for all patients to receive regular video assessments and to only be invited for injection when deemed necessary based on the tele-assessment. Here, the COVID-19 pandemic may lead to a much-needed technological “push”.

#### 2.2.2. Staff Shortages and Training

Staff shortages are prevalent due to absence from work because of COVID-19 infections or burn-out [20], and due to self-isolation in case of personal risk, caring for children at home as schools and childcare facilities are closed, or quarantine. Additionally, toxin services often deal with a lack of trained and experienced physicians capable of delivering BoNT injections. This was already a problem before the pandemic, due to increasing demand. Now, the issue is exacerbated because some training programs were suspended and treatment sessions must be performed as quickly as possible, leaving insufficient time for hands-on training and demonstrations for junior physicians. When the pandemic ends, an increased number of patients will request consultations and the lack of experienced injectors may lead to long and unacceptable treatment delays. In our opinion, it would be useful to establish web-based training courses (webinar lectures, seminars) and case discussions in the field of BoNT treatment for focal dystonia, focal and multifocal spasticity, and sialorrhea, to increase interested physicians’ level of knowledge in this field. This should allow for increased theoretical knowledge in young physicians in a motivating atmosphere and increased preparation for starting hands-on training. It may even be feasible to teach the method of ultrasound-guided injections with, e.g., a two-camera or channel system showing the position of the probe and the ultrasound screen for BoNT injections into small or deep-seated muscles. However, this will require hands-on training when the pandemic allows. Online support for junior colleagues is also useful, with senior physicians providing advice on patient evaluation, muscle injection strategy, orthotic management, difficult cases, and trouble shooting. We envisage that, due to the COVID-19 pandemic, tele-medicine methods will feature much more prominently in future training curricula for medical students and junior physicians [19].

#### 2.2.3. Visiting the Patient in Community Settings

It has been suggested that toxin services could be shifted to be delivered in “community settings”, i.e., at home, in care facilities (domicile treatments), and in community clinics. Obviously, this is only feasible in countries where national regulations allow it. Such treatments outside of the hospital setting can protect patients from potential SARS-CoV-2 exposure in transport, hospitals, and rehabilitation centers. In our opinion, treating patients at home should generally be an option for a physician, if necessary, and also for non-pandemic-related reasons. We have some experience using pocket-sized ultrasound, electrical stimulation or electromyographic devices that the physician can use to administer guided toxin injections outside the hospital setting.

### 2.3. Optimizing Efficacy and Safety of Treatments

Efficacy, safety, and dosing regimens for BoNT treatments have been extensively studied over the last decade [7,21,22,23]. However, the question is what impact the pandemic may have on these aspects and how modern technology can be utilized.

#### 2.3.1. The Treatment Session and Dosing Considerations

Dose selection procedures and the selection of muscles to be injected have not changed. Now, most importantly, the injection session must be kept as short as possible, to reduce contact times between patient and staff to a minimum, and appropriate PPE is essential.

Another newly emerging aspect is the question of temporal spacing between a COVID-19 vaccination and a BoNT injection. While strong data on this are not available yet, experts agree to err on the side of caution. In our opinion, the two types of injections should not be administered on the same day, if possible, to differentiate between acute reactions arising from the vaccination and side effects of the BoNT injection. This approach has been backed up by medical experts consulting in clinical trials that we are involved in. From a pharmacological point of view, we assume no direct interaction between the vaccine compounds and BoNT, but we cannot know for certain at this time.

With regards to dosing, we agree with other experts that it should be kept at the necessary level for each patient, as before the pandemic [24]. Any treatments missed due to the pandemic do not require compensation by injection of increased BoNT doses in the next injection session, unless symptoms or limitations are more severe than usual. Gaps in treatment also occurred in previous years, due to sickness, difficulties scheduling an appointment, holidays, etc. As before the pandemic, a patient should be assessed carefully before an injection and the suitable dose determined based on the presented clinical status. A survey from Italy assessed patients’ first BoNT doses after a mean treatment delay of 2.4 months due to lockdown measures. Here, doses were not significantly different from those administered to a control group of patients who managed to adhere to their usual treatment schedule [25].

To generally prolong treatment intervals, a case has been made for modest increases in doses, albeit only if previous doses were in the lower range of the recommendations for the targeted muscles. This can allow patients to visit the hospital less frequently, thus minimizing the risk of exposure to SARS-CoV-2. In the literature, several recent publications suggest that higher incobotulinumtoxinA doses (>400 to 840 units) are safe and well-tolerated [22,26,27,28,29], while others do not rule out increased risks of systemic side effects [30,31,32,33]. Therefore, dosing has to be considered carefully by the treating physician, on a patient-by-patient basis, and must take into account the previously used dosing levels to assess how many doses could be increased without undue risk.

The question has been raised in the medical community as to whether toxin treatments may increase susceptibility to SARS-CoV-2 infection, i.e., whether treatments should be suspended, or doses lowered during the pandemic. It has been argued that toxins may lead to muscle weakness and this may result in respiratory problems, making patients more susceptible to COVID-19 and its complications. We agree with other experts that this is not the case. If BoNT is administered correctly and under instrumental guidance as recommended [5,34,35], the risk of muscle weakness events is very low, even in areas near the respiratory muscles. The possibility of spasticity/dystonia symptoms or sialorrhea worsening to unacceptable levels presents a much higher risk to patients’ health status.

#### 2.3.2. Tele-Communication Support

Tele-communication tools, particularly video conferencing, can help assess patients’ symptoms, side effects, and re-treatment needs [2,10,17,18,19]. If a patient’s status is satisfactory as seen/discussed by video call, the re-treatment interval can be increased. Ideally, every patient would receive re-treatment only when necessary, at individually set intervals after video consultation. This can optimize efficacy and safety at the same time. Obviously, a full muscle tone assessment for spasticity/dystonia patients is not possible by video call. However, a number of assessments are possible, also with the help of the carer, depending on the patient’s situation [36]. Muscle tone does not have to be the focus; the main goal should be to assess the impact of muscle tone on patients’ and carers’ lives.

The downside of video communication is the potential decrease in personal contact and face-to-face visits, and the resulting impact on the relationship between patient and physician. This must be kept in mind and video communication needs to be used wisely by dedicated and trained staff to minimize such effects [19,37]. Simple telephone calls are an alternative way of communicating with elderly and/or severely disabled patients who cannot handle video technology, or when internet connections are not sufficient [38].

The most pressing issue is the urgent need for a wider availability of certified video communication systems that comply with data protection rules in each country and are reimbursed. Currently, many physicians use publicly available commercial video call platforms, which poses data protection issues when used to discuss a patient’s health status and treatments. We urge colleagues to find out what is available in their countries and use the secure technologies that are on offer. The sector is currently expanding at unprecedented speed, with the increased demand for tele-medicine tools due to the pandemic [11,39]. Where no tools are officially endorsed, colleagues should actively demand support from national health care providers.

The National Health Service (NHS) in the UK set an example by offering access to communication software and an app for physicians and staff via the NHS communications system, allowing secure video conferencing among staff and with patients [40,41]. The German Medical Association also released recommendations for software tools and for reimbursement of video consultations [42].

An ideal communication platform is CE-certified as a medical device and reachable by smart phone or tablet. It can be used to provide training, advice, and exercise demonstrations, and allows objective measurements to assess and follow-up changes in a patient’s condition. It also allows the monitoring of symptoms and of adherence of patients to the proposed treatment or exercise routines. The use of such platforms must be approved and paid for by insurers and national health systems in the same way as face-to-face support. While such technology is still not readily available everywhere, we would like to stress the importance of this for bridging the gap in patient care during the pandemic, but also to generally optimize patient care and treatment efficacy by using technology to support patients between visits. We expect long-term benefits of this development, independent of the pandemic. With more technological support, patients can visit their hospital/clinic less frequently, allowing the facility to offer face-to-face appointments to other patients in need. This would give more patients access to toxin treatments and make flexible treatment interval management more feasible. Furthermore, less frequent visits to the facility results in lower transport costs and fewer days of absence from work/school for patients and carers.

The pandemic may give a long-needed boost to efforts to modernize injection scheduling and symptom tracking, to better educate patients and carers, to assess their needs, and to manage the patient’s condition at home.

## 3. Discussion and Conclusions

For this paper, we evaluated the experiences from ToxNet group members in order to arrive at the best recommendations and practices that we think can be implemented at most facilities, despite their differences. The overall aim must be to provide adequate patient-centered care and timely multimodal spasticity/dystonia/sialorrhea management to patients, as well as protecting patients and staff from SARS-CoV-2 exposure. While the peak of the pandemic appears to have been reached in some countries, the emergence of virus variants around the globe and the current uncertainty as to the long-term effectiveness of the vaccines will mean that certain protective measures are here to stay. As one commentary put it, “no country can be safe until all countries are safe” [43].

We would like to view the COVID-19 pandemic not only as a crisis with a huge socio-economic impact on healthcare services and on patients’ health globally, but also as an opportunity. As in other sectors, the changes and new measures that had to be implemented in toxin services due to the pandemic can now be fine-tuned and adjusted into a “new way of working”.

## 4. Materials and Methods

This paper was developed by experts from the ToxNet group (www.toxnet.net, accessed on 21 August 2021), originally based on a virtual round table held in July 2020. At this meeting, the impact of the COVID-19 pandemic on toxin services and strategies for dealing with the situation were discussed and opinions were collected. In addition to the ToxNet experts leading the discussions, an online audience of approximately 200 attendees participated in the event and could post questions and suggestions. After the round table meeting, further discussions followed, as the pandemic developed. The relevant literature from 2020 and 2021 was consulted, to arrive at these recommendations for toxin services.

The ToxNet group was initiated in 2016 and is a global network of leading experts and specialists in the field of neurological movement disorders rehabilitation. The group consists of 21 experts practicing in 14 countries, 7 of whom are well-known international key opinion leaders. With a collective experience of more than 250 years (range 4–25 years; average 14 years), the group is dedicated to advancing the science of BoNT treatment in movement disorders. Some of ToxNet’s published works to date include a consensus on optimization of post-stroke spasticity interventions with BoNT, an evaluation of spasticity patients using telemedicine, and an opinion paper on the role of physical and rehabilitation medicine in the COVID-19 pandemic [5,36,44].

## Figures and Tables

**Table 1:** Patient risk assessment regarding their toxin treatment urgency.

Assessment	Recommended Stratification
How urgently does the patient require toxin treatment for his/her neurological condition? Is it justifiable to postpone treatment?	Schedule a video call (if possible) or a phone call to stratify patients according to several aspects *:
	**High urgency** acute increase in pain level and/or acute increase in symptoms (fast deterioration within the last 4 weeks), significant interference with usual activities/roles patient or carer requests treatment urgently ▶ Inject now: call patient in for injection, should not be postponed. ▶ If/when available: perform rapid on-site test for SARS-CoV-2 upon arrival.
	**Medium urgency** no acute changes in pain level or symptoms existing pain or symptoms manageable with medication and exercises at home patient or carer may be willing to postpone treatment ▶ Treating physicians and team must discuss in detail with patient or carer whether postponing injection is justifiable. ▶ If SARS-CoV-2 risk assessment results in low risk, call patient in for injection. ▶ If SARS-CoV-2 risk assessment results in high risk, provide patient with guidance and coping strategies by phone or video call, send medication prescriptions, postpone injection, and review risk again 4 weeks later.
	**Low urgency** low pain level, only few symptoms symptoms manageable with medication and exercises at home patient or carer asks to postpone treatment ▶ Watch and wait: injection can be postponed. ▶ Provide patient with guidance and coping strategies by phone or video call, send medication prescriptions. ▶ Schedule next risk review in 4 weeks’ time.

* Note: Consider whether these aspects were rated by the patient himself/herself or by the carer, and to what extent an acute fear of SARS-CoV 2 infection may influence patient’s/carer’s responses.

## Data Availability

Not applicable.
